# Photodynamic inactivation of *Botrytis cinerea* by an anionic porphyrin: an alternative pest management of grapevine

**DOI:** 10.1038/s41598-020-74427-9

**Published:** 2020-10-15

**Authors:** Veronica Ambrosini, Mohammad Issawi, Vincent Sol, Catherine Riou

**Affiliations:** PEIRENE-EA7500, Faculty of Sciences and Technology, University of Limoges, 123 avenue Albert Thomas, 87060 Limoges, France

**Keywords:** Plant sciences, Plant stress responses, Biotic

## Abstract

*Botrytis cinerea* is a necrotic plant fungus that causes gray mold disease in over 200 crops, including grapevine. Due to its genetic plasticity, this fungus presents strong resistance to many fungicides. Thus, new strategies against *B. cinerea* are urgently needed. In this context, antimicrobial photodynamic treatment (APDT) was considered. APDT involves the use of a photosensitizer that generates reactive oxygen species upon illumination with white light. Tetra-4-sulfonatophenyl porphyrin tetra-ammonium (TPPS) was tested on *B. cinerea* using light. 1.5 µM TPPS completely inhibited mycelial growth. TPPS (12.5 µM) was tested on three grapevine clones from Chardonnay, Merlot and Sauvignon, grown in vitro for 2 months. Treated root apparatus of the three backgrounds increased thiol production as a molecular protection against photoactivated TPPS, leading to a normal phenotype as compared with control plantlets. Finally, 2-month-old grapevine leaves were infected with 4-day-old mycelium of *B. cinerea* pre-incubated or not with TPPS. The pre-treated mycelium was unable to infect the detached leaves of any of the three grapevine varieties after 72 h growth when subjected to a 16 h photoperiod, contrary to untreated mycelium. These results suggest a strong potential of photo-treatment against *B. cinerea* mycelium for future agricultural practices in vineyard or other cultures.

## Introduction

The great challenge of agriculture is to produce sufficient food for the ever-growing world population. Since the 60 s, to access this performance, agri-business practices that include excessive uses of pesticides and fertilizers are becoming the main cause of soil, water and air pollution, as well as loss of fauna and flora biodiversity^1,2^. Moreover, this industrial agriculture triggers major public health problems such as infertility, cancers and child malformations^3,4^. Aware of the need to reduce all these dramatic environmental damages, the European Union introduced the directive 2009/128/EC to reduce the use of pesticides.

To fight off plant competitors and pathogens, new approaches are necessary for safe practices in agriculture such as genetically modified plants, genetic improvements, as well as organic and integrated agriculture^5,6^. The photodynamic treatment is a general and new concept with a large spectrum of applications for animal and plant cells, plant and animal pathogens, as well as microorganisms^7–18^. Thus, photodynamic treatment could represent an innovative and powerful strategy to fight off plant competitors and pathogens in future agricultural practices^17^. One of the key actors of APDT is a molecule called a photosensitizer (PS). When irradiated with light, this molecule produces reactive oxygen species that are toxic for cells^19^. By contrast, most PSs present low levels of cytotoxicity or genotoxicity in the dark^9,20^. Furthermore, biological applications are best conducted with water-soluble PSs which are ideally prone to quick photodegradation, thus avoiding a buildup of toxicity. PSs are classified in many groups such as porphyrins, chlorins, coumarins, furocoumarins, phthalocyanines and phenothiaziniums. Porphyrins and chlorins such as chlorophyllin, have been shown to be very effective against bacteria on kiwi leaves tested in vitro^21,22^. Coumarins, furocoumarin and phenothiaziniums were shown to be active against the plant-infecting fungi *Colletrichum acuratum* and *Aspergillus nidulans*^23–25^. Finally, when tested on *Citrus sinensis* petals and leaves, methylene blue was able to kill the *Colletotrichum abscissum* fungus and proved to be harmless to plant organs; in addition, this treatment did not induce any secondary resistance^26^.

Previous research works explored the in vitro phenotypical and molecular responses of Arabidopsis and tomato plantlets to the photodynamic stress induced by an exogenous supply of PS^27,28^. The cationic tetra (N-methylpyridyl) porphyrin, either free base or zinc-complexed, tested at 3.5 µM, inflicted harmful effects on both 14-day-old Arabidopsis and tomato plantlets. Nevertheless, while Arabidopsis plantlets were killed, tomato plantlets could be rescued after a 14 day-treatment^28^. Surprisingly, the anionic porphyrin tetra-4-sulfonatophenylporphyrin tetra-ammonium (TPPS) did not provoke any harmful effect on both plantlets even at concentrations as high as 50 µM^27,28^. With the aim to develop APDT for agriculture applications, TPPS could be a good candidate because of its low toxicity for plants. Moreover, TPPS remains negatively charged in many chemical environments even under acidic pH and does not aggregate in solution, allowing it to easily permeate cells through cell walls and membranes^29,30^. Therefore, we hypothesized that TPPS could be a good PS candidate to kill the plant pathogen *B. cinerea* and has potential to be a safe option for grapevine (*Vitis vinifera* L.) explants.

*B. cinerea* is frequently responsible for drastic reductions in crop yields at harvest and for reducing wine quality^31,32^. This fungus displays very strong resistance to many fungicides, due to its genetic plasticity which confers its diversity in morphology, mycelial growth, sporulation and virulence^33–37^. For the Integrated Pest management, the grapevine’s susceptibility to *B. cinerea* can be considered an essential management indicator^38–43^. According to the classification proposed by Dubos^42^ and Fermaud et al.^43^, Chardonnay and Sauvignon are highly susceptible to *B. cinerea* infection whereas the Merlot variety is more resistant. Furthermore, these three varieties are listed in the top 10 most cultivated and famous grapevines for wine production in the world^44^.

Therefore, as a first and necessary step, TPPS was tested separately on the three grapevine backgrounds and on *B. cinerea* mycelium with the aim of killing the plant pathogen without affecting plantlet growth and development. As a second and final step, grapevine detached leaves infected with *B. cinerea* mycelium were tested with and without TPPS pre-treatment.

## Results

### Mycelium growth

**Figure 1:**
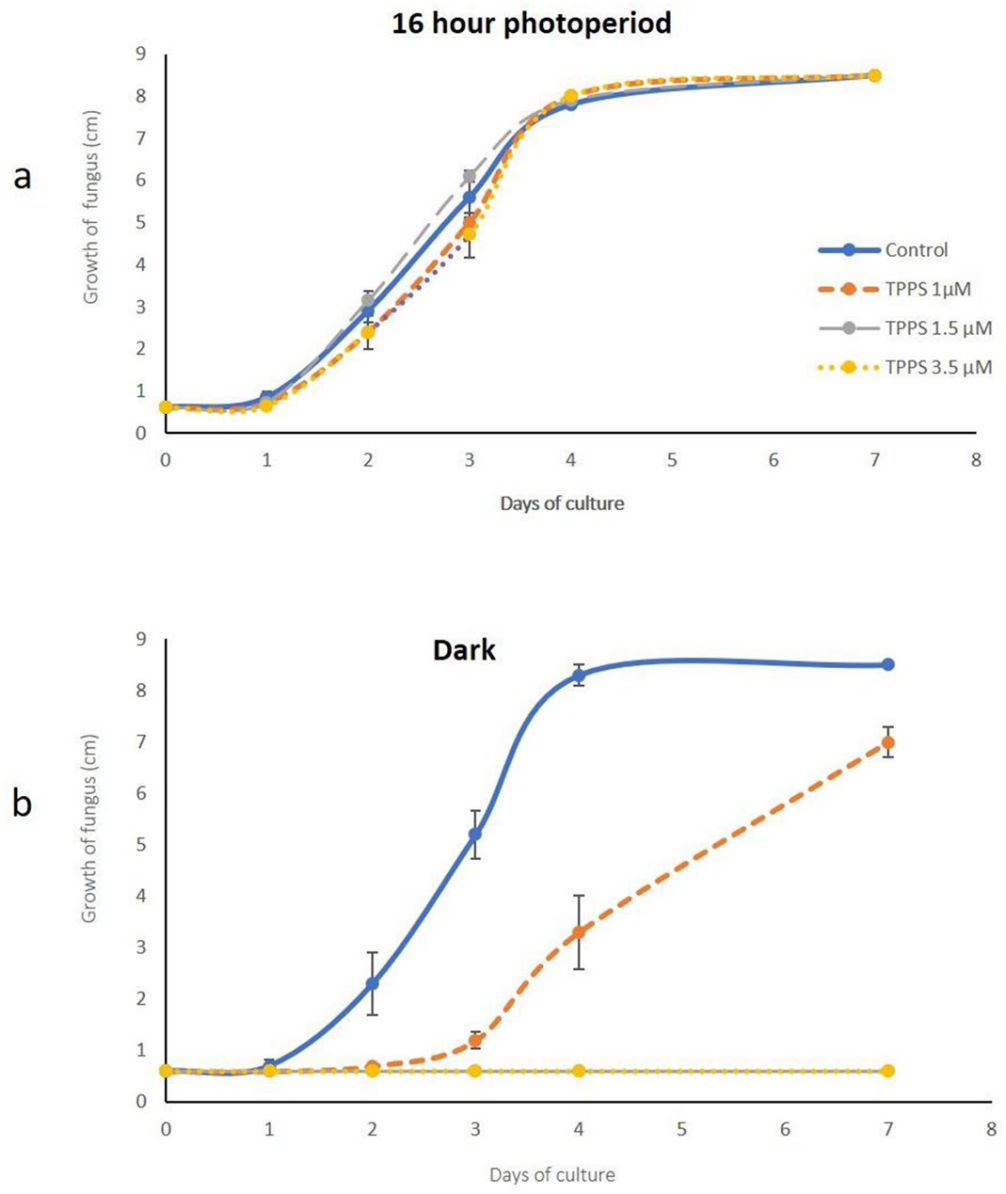
Growth curves of *B. cinerea* mycelium (**a**) in the dark conditions and (**b**) under 16 h photoperiod. Growth curve of *B. cinerea* was performed as follows: a plug of 0.6 cm diameter was placed in the middle of plates containing PDA medium supplemented with or without TPPS. Three TPPS concentrations: 1, 1.5 and 3.5 µM were tested in the dark and under light. Results are the mean of three independent experiments ± sd.

The effect of three increasing concentrations of TPPS was monitored on *B. cinerea* mycelium growth under dark and light conditions as described in the material and methods section. As the four curves obtained with or without TPPS in the dark coincided within the standard deviation, we confirmed that, by itself, TPPS did not exhibit any cyto- and/or genotoxic activity against the fungus (Fig. 1a). Under light and at a low TPPS concentration (1.5 µM), mycelium growth was completely inhibited (Fig. 1b). Furthermore, 1 µM TPPS significantly slowed down mycelium growth under light; however, after a 7 day-culture, the colony reached a size similar to that of the control (Fig. 1b). Therefore, 1.5 µM of photoactivated TPPS has been chosen as the minimum fungicidal concentration (MFC). As shown by the growth curves in the dark and in light conditions, the fourth day of culture corresponded to the end of the exponential growth phase (Fig. 1). Thus, all further experiments were conducted with 4-day old mycelium.

### Effect of photoactivated TPPS on hyphae morphology

**Figure 2:**
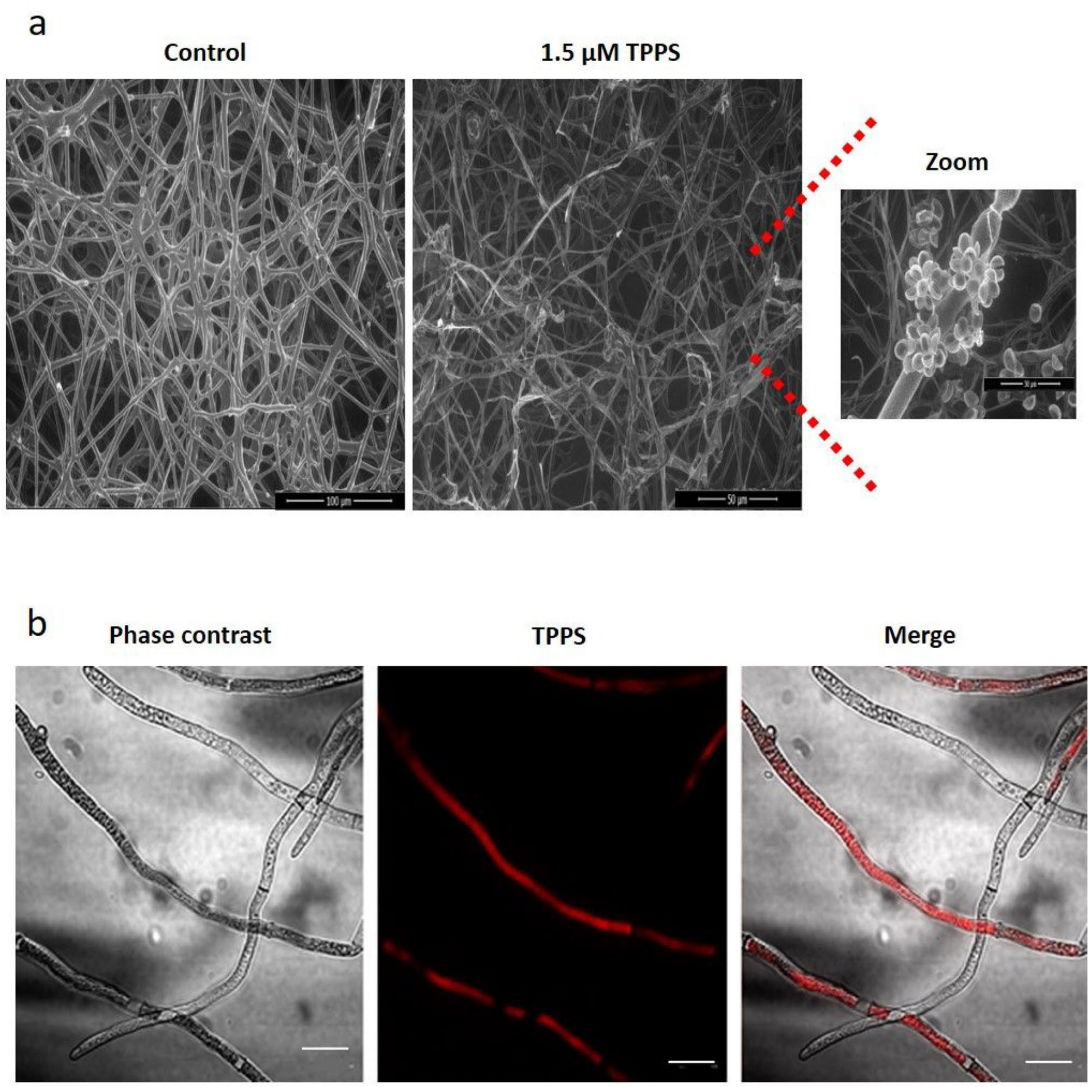
Microscopic observation of *B. cinerea* and TPPS localization in mycelial cells. (**a**) *B. cinerea* visualized under light using environmental scanning electronic microscope. The left picture corresponds to the control hyphae and the middle picture to the irradiated hyphae treated with 1.5 µM TPPS. Treated hyphae shows spore formation (right image). (**b**) TPPS localization in *B. cinerea* hyphae by confocal microscopy. *B. cinerea* was cultivated for 3 days in presence of 3.5 µM TPPS in the dark. Sample was excited at 405 nm and TPPS detection was performed under spectral acquisition with a peak of emission around 640 nm. Scale bar: 20 μm.

As the mycelium growth was affected by photoactivated TPPS, it was decided to carefully look at the hyphae structure, using ESEM. As expected, photoactivated TPPS induced important phenotypic changes of the hyphae, compared with the control that showed very regular hyphae with a well-organized structure (Fig. 2a). In presence of 1.5 µM TPPS, hyphae were notably less organized, exhibited irregular shapes and produced some spores (Fig. 2a). Moreover, the TPPS-treated hyphae presented a reduced width (1.36 µm) compared with the control (4.7 µm) (data not shown).

### TPPS localization inside mycelial cells

According to the phenotypical effects observed in TPPS-treated mycelium under light, it was of interest to localize TPPS inside the cells. TPPS was found inside several cells of 4-day-old mycelium (Fig. 2b). This intracellular localization could explain the very strong inhibitory effect of photoactivated TPPS on mycelial growth. As TPPS was located inside cells, this showed that it could cross the cell wall and accumulate in the cell cytoplasm.

### Biochemical activities of TPPS-treated *B. cinerea* mycelium

**Figure 3:**
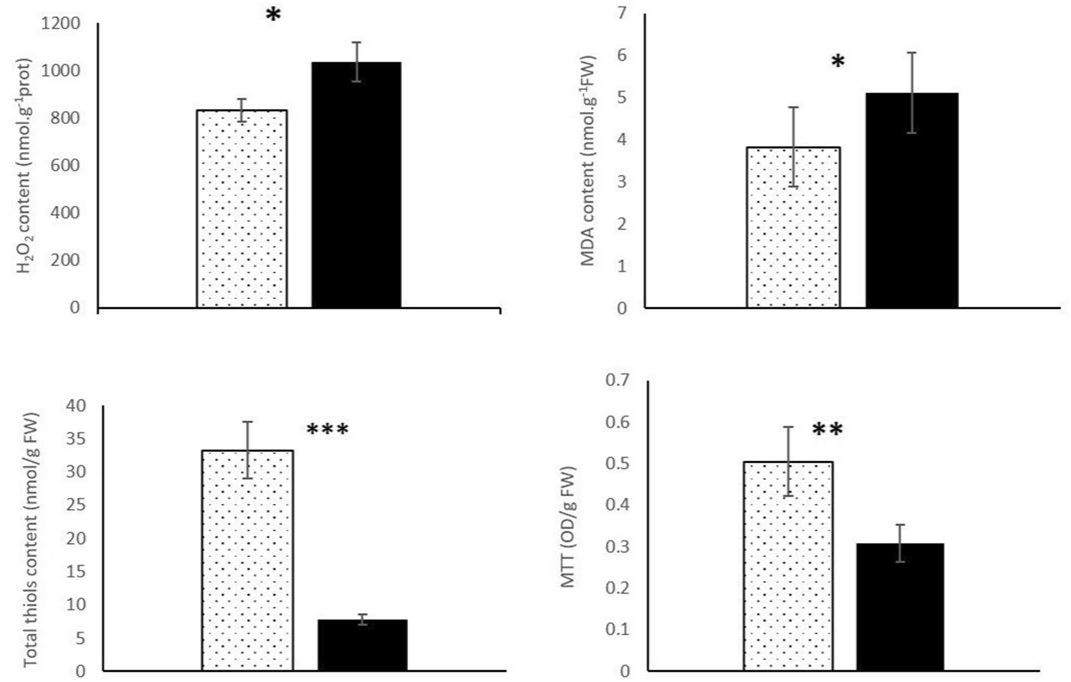
Biochemical activities measured in *B. cinerea* mycelium under a 16 h photoperiod white light for 4 days. Hydrogen peroxide (H_2_O_2_) production_,_ MDA, total thiol contents and MTT assay were performed with control mycelium (untreated, dotted box) and mycelium treated with 1.5 µM TPPS (black box). Results are the mean of three independent experiments. Statistical significance is determined by a one way ANOVA test (*P < 0.05, ***P < 0.001).

To gain insight into the effect of photoactivated TPPS on the mycelium, biochemical assays linked to oxidative stress and cell metabolism activity, were conducted. As expected, increases in both H_2_O_2_ and MDA contents were observed in treated mycelium compared to the control, demonstrating at the molecular level that the fungus was stressed (Fig. 3). Metabolic activity, linked to mitochondrial respiration, was monitored with the MTT assay. Indeed, under light, formazan production decreased in the treated hyphae compared with the control, thus explaining the fungal growth inhibition (Fig. 3). While MDA indicated an increase in lipid peroxidation in the presence of photoactivated TPPS, the thiol content provided additional evidence that the fungus was not dead but only deeply stressed (Fig. 3).

### TPPS and grapevine plantlets grown in vitro

**Figure 4:**
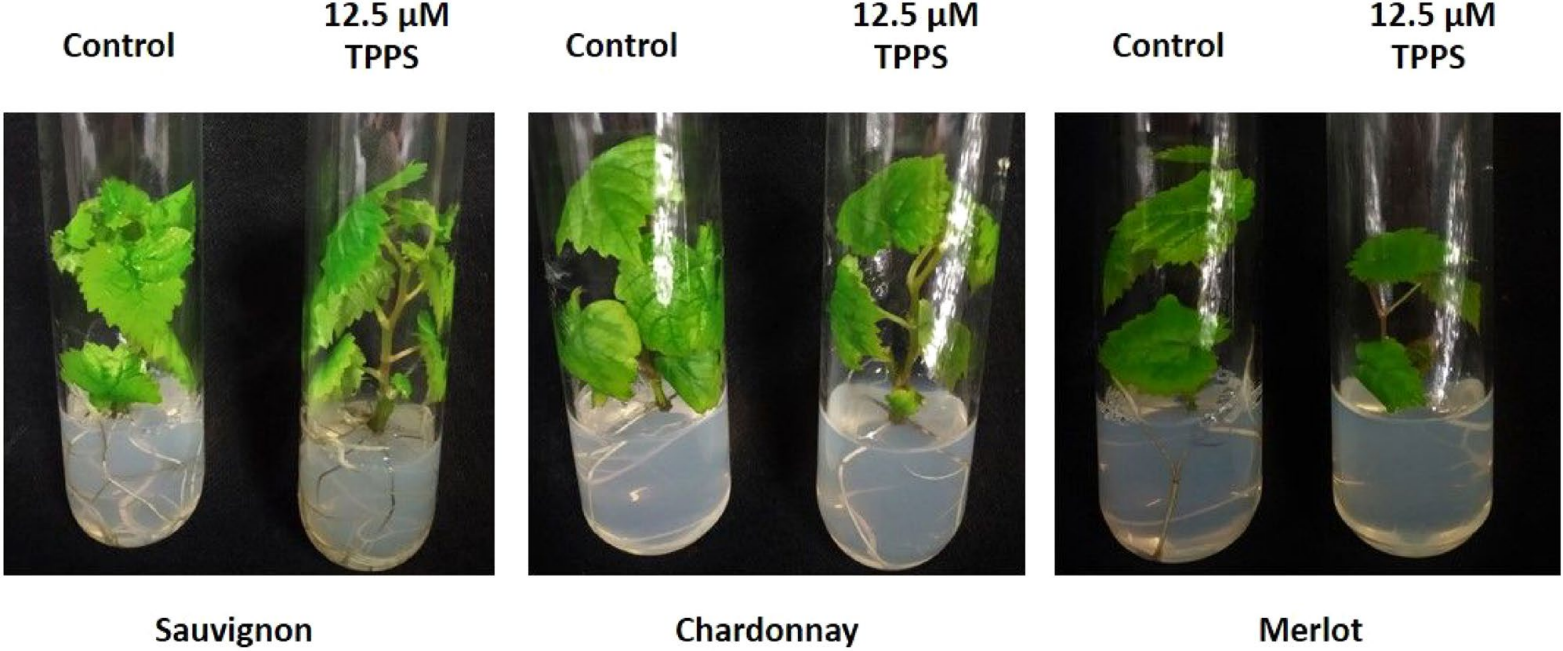
Grapevine varieties growing on control medium and on medium supplemented with 12.5 µM TPPS. Pictures correspond to plantlets from each variety, cultured during one month in glass tubes containing control medium (left) or medium with 12.5 µM TPPS (right).

Internodal explants from each variety: Merlot, Sauvignon and Chardonnay, were placed on 12.5 µM of TPPS for two months in growth chamber as described in the material and methods section. Each culture was examined during a period of 3-months and the explant sub-culture was performed from 2-month-old plantlets. There was no phenotypical difference between the control and the treated plantlets after a 1-month culture (Fig. 4). To confirm the absence of the phenotypical effect of photoactivated TPPS, we measured the thiol content in aerial and root organs of treated and control plantlets from the three varieties (Table 1**)**. Roots from the three varieties, that were in contact with photoactivated TPPS, showed a significant increase in the total thiol content compared to the control (Table 1). At the aerial level, while no difference in thiol content was observed in Sauvignon and Merlot, there was a significant difference in the total thiol content measured in Chardonnay (Table 1). This could suggest that the Chardonnay variety is more sensitive to photoactivated TPPS than the two other backgrounds although no visible outcome could be seen at the phenotypic level (Fig. 4).

**Table 1:** Total thiol content of 2-month old grapevine plantlets.

	Total thiol content (nmol/g FW)			
	Roots		Aerial organs	
	Control	TPPS	Control	TPPS
Sauvignon	66.63 ± 17.96	134.12 ± 44.44**	174.8 ± 42.13	246 ± 55.46
Chardonnay	44.47 ± 5.8	77.5 ± 14.8**	69.35 ± 11.18	106.7 ± 13.4*
Merlot	107.7 ± 14.56	361.38 ± 55.45**	292.77 ± 60.9	297.7 ± 70.3

Roots and aerial organs from each variety: Sauvignon, Chardonnay and Merlot, were separately frozen and analyzed. TPPS was tested at 12.5 µM. *FW* fresh weight. Results are the mean of three independent experiments ± sd. Statistical significance is determined by a one way ANOVA test (*P < 0.05, **P < 0.01). Statistical analysis was always performed against the control. When no indication, not significant.

### TPPS effect on *B. cinerea* infected leaves

**Figure 5:**
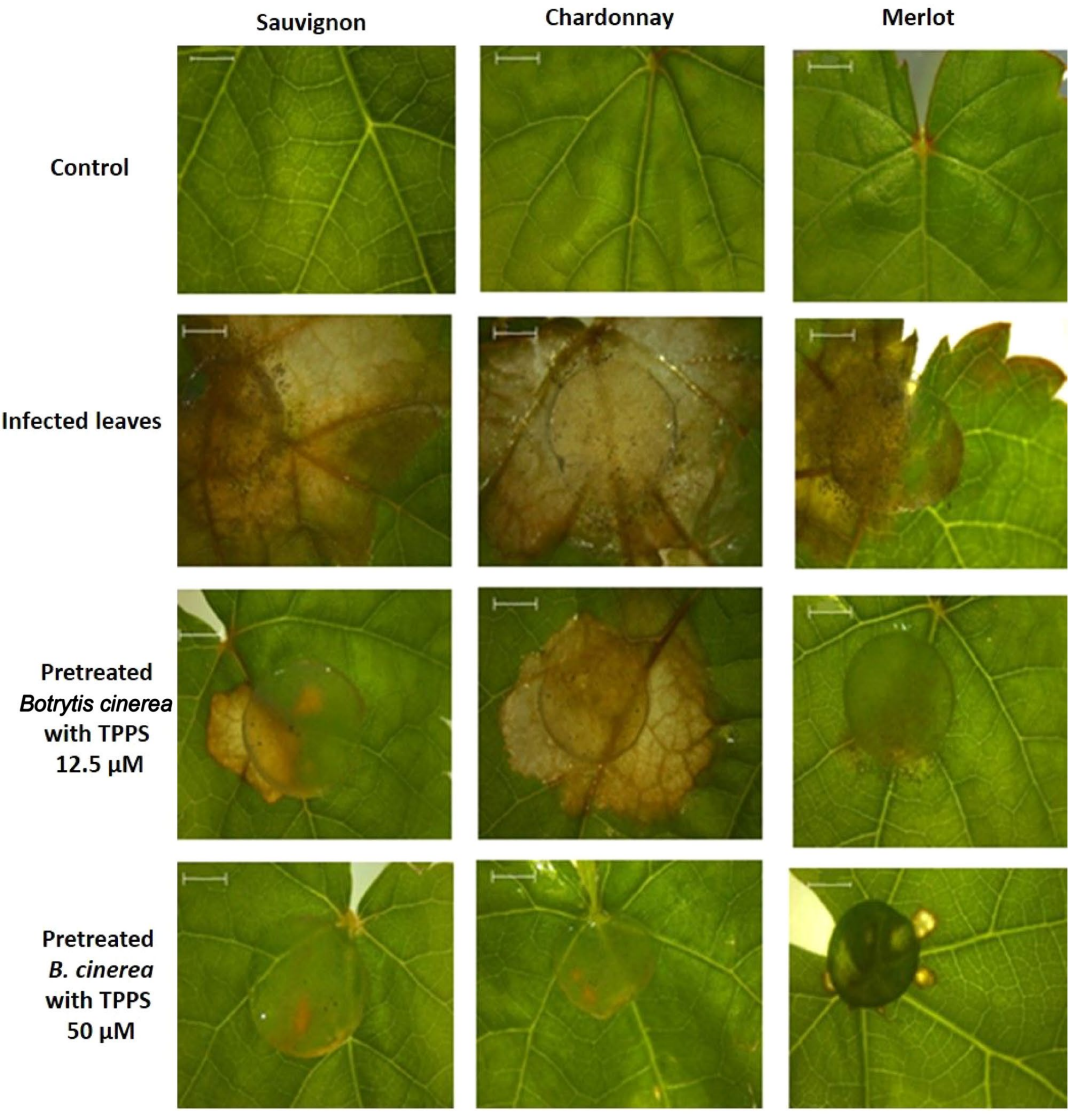
TPPS pre-treatment of *B. cinerea* leads to inhibition of mycelial growth on grapevine leaves. Pictures were taken after a 72 h infection of *B. cinerea*. Detached leaves from the three varieties, without any contact with *B. cinerea* (upper panel). On the three other panels, 4-day-old *B. cinerea* mycelium plugs were placed on detached leaves. Before contact with these leaves, the plugs were pre-incubated or not with 12.5 or 50 µM TPPS for 8 h under dark conditions. Subsequent to pretreatment with 12.5 µM TPPS, mycelium growth was reduced, but the infection was not inhibited. The lower panel corresponds to mycelium pretreatment with 50 µM TPPS: *B. cinerea* was no longer able to invade the leaf surface. The circular plugs correspond to the 6 mm mycelium disc placed on the leaf surface at the beginning of the experiments. Scale bar: 2 mm.

The final interest of this work was to put together grapevine, *B. cinerea* and TPPS with the expectation to kill the pathogen without disturbing plants. As a preliminary assay, before the development of a complete plant pathosystem, our antifungal photodynamic treatment was tested on detached grapevine leaves from two-month-old plantlets. Experiments were conducted as described in the material and methods section, in the growth chamber. The infection was monitored for 72 h. Before 48 h, no change was observed in leaves from the three clones (data not shown). After 48 h, the untreated *B. cinerea* started to invade the leaf surface of the three varieties. The growth of the mycelium, pre-treated with 12.5 µM of TPPS for 8 h, was not completely inhibited (Fig. 5). However, after a 50 µM TPPS pre-treatment, the growth of mycelium was totally inhibited on the leaf surface suggesting that the strategy described in this study worked efficiently.

**Figure 6:**
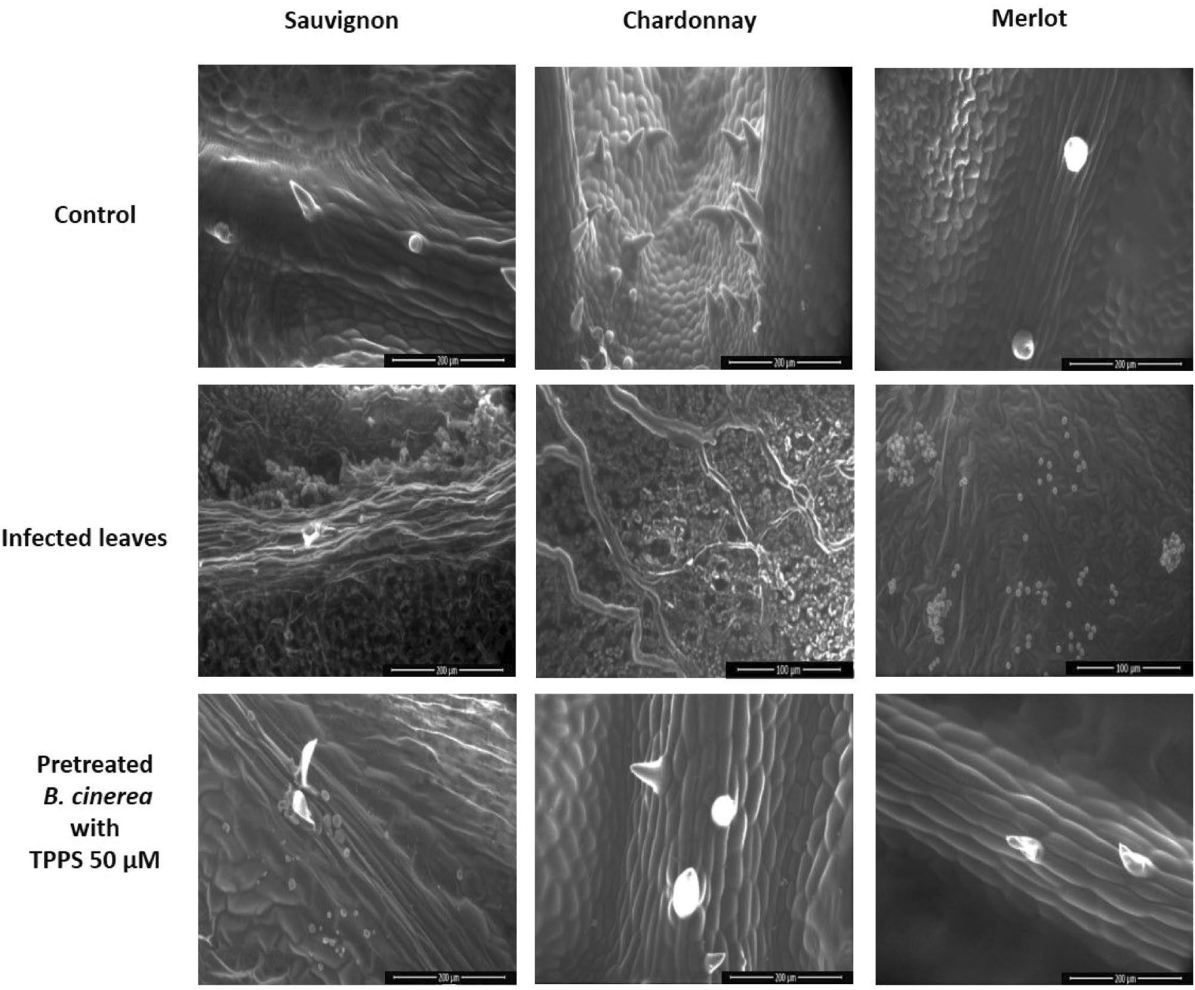
Scanning microscopy observations of leaves infected or not by *B. cinerea* pretreated or not with TPPS. After treatment with TPPS, the fungus was unable to infect the Chardonnay and Merlot leaves. For the Sauvignon variety, spores have been observed on leaf surface, even after TPPS pretreatment.

ESEM analysis was conducted on infected leaves. In uninfected leaves, the structure was well-defined and organized (Fig. 6). However, in the infected leaves when *B. cinerea* was not pre-treated with TPPS, the fungus completely invaded the leaves, making the structure unrecognizable (Fig. 6). By contrast, when *B. cinerea* was pre-treated with 50 µM of TPPS, ESEM pictures showed a leaf structure almost identical to that of the control confirming the macroscopic phenotype (Fig. 6). It was noticed for the Sauvignon variety, that spores were present in the pre-treated *B. cinerea* suggesting that Sauvignon was more susceptible than the other two varieties.

For further investigation, biochemical assays were performed. H_2_O_2_ quantification, linked to oxidative stress, was conducted for the three leaf conditions and the three varieties after infection or not with TPPS. After a 72 h treatment, a basal content of H_2_O_2_ was detected in the three leaf varieties, very similar for Chardonnay and Sauvignon leaves and slightly lower for Merlot (Table 2). When the leaves were infected by a 4-day-old *B. cinerea* mycelium, the H_2_O_2_ content significantly increased for the three varieties especially for Sauvignon (more than 3.5-fold higher than the control leaf) explaining its strong susceptibility to *B. cinerea* (Table 2). An increase in H_2_O_2_ production in all leaf types in contact with *B. cinerea* was expected. Concerning the leaves infected by the fungus pre-treated with 50 µM TPPS for 8 h, no significant difference was observed between Chardonnay and Merlot leaves compared to leaves infected by the fungus. No signs of infection were observed after 72 h culture (Fig. 5, Table 2). *B. cinerea*, pre-treated with TPPS, was no longer able to induce a high production of H_2_O_2_ in Sauvignon leaves (Table 2). The H_2_O_2_ content nearly reached the basal level detected in the control Sauvignon leaves (Table 2). As a response to this H_2_O_2_ production detected in the leaves induced by *B. cinerea*, and to gain insight into the leaf’s proper response, we measured the total thiol content in leaves that were subjected to the three different treatments. For Chardonnay and Merlot detached leaves, no significant difference was observed between the control, infected leaves and infected leaves pre-treated with TPPS (Table 2). A significant increase in thiol content was only observed for Sauvignon leaves between the control and the infected leaves. This result suggested that Sauvignon leaves were able to fight against the fungus infection with a thiol induced response (Table 2). Nevertheless, we also confirm that our pretreatment with TPPS inhibited *B. cinerea* growth on leaves for each variety. These results are promising for the development of APDT treatments in agriculture (Figs. 5, 6).

**Table 2:** Biochemical activity assays in detached grapevine leaves infected or not by *B. cinerea*.

	Hydrogen peroxide content (nM g^−1^prot)			Total thiol assay (nmol/g FW)		
	Control (C)	Infected leaves (IL)	Pretreated *B. cinerea* (P*Bc*)	Control (C)	Infected leaves (IL)	Pretreated *B. cinerea* (P*Bc*)
Sauvignon	255 ± 61	904 ± 99 C = ***	338 ± 88 IL = ***	337 ± 44 IL = *	434 ± 34	340 ± 41 IL = *
Chardonnay	204.6 ± 59	473 ± 86 C = ***	408 ± 57 C = ***	206.6 ± 41	291.6 ± 58.3	351.3 ± 59.5
Merlot	156 ± 35	396 ± 26 C = **	293 ± 68 C = *	282 ± 25	323.4 ± 58	259.5 ± 9

Detached leaves from Sauvignon, Chardonnay and Merlot varieties were tested and analyzed.

*C* control (healthy leaf), *IL* leaves infected with *B. cinerea*, *PBc* leaves infected with *B. cinerea* pre-incubated with 50 µM TPPS for 8 h, *FW* fresh weight.

Results are the mean of three independent experiments. Statistical significance is determined by a one way ANOVA test (*P < 0.05, **P < 0.01; ***P < 0.001). Statistical analysis was performed for each assay and was represented as follows: C = *** in IL column meant C compared to IL with a P < 0.001. No indication means not significant.

## Discussion

*Botrytis cinerea* is a very serious problem in a large variety of plants. It is a necrophytic fungus that induces ROS production that contributes to plant cell destruction during its infection^45^. This fungus is able to infect leaves, stems, flowers and fruit, causing severe damages and commercial losses in agriculture. In vineyards, the fungus induces several deleterious effects on both quality and quantity of vine production. Despite all the damage it can cause, under specific weather conditions, its growth on grapes induces noble rot that gives rise to sweet wine. However, the fight against this pathogen remains a daily struggle especially for fruit production in summer or fall. For more than 50 years, the use of specific fungicides has largely been envisaged and in that time, *B. cinerea* found coping strategies. As a consequence, fungicide treatments gradually became inefficient, even the famous CuSO_4_ solution also known as ‘Bordeaux mixture’ lost its effectiveness against fungal pathogens^46,47^. Moreover, the copper divalent ion, also toxic for plants, contributes to soil pollution^48,49^.

Therefore, new strategies against fungi are urgently needed. Wang et al.^50^ used naturally occurring eugenol (EC50 value of 235 µM for *B. cinerea*). This was found to mainly affect fungal mycelium growth rather than the germination of spores as shown in previous reports on fungicides, such as carbendazim and *N*-phenyl carbamates^51,52^.

In a similar way, Fleurat-Lessard et al*.*^53^ discovered a strategy whereby they investigated the potential of FeSO_4_ and found that the sulfate anion determines the inhibition of mycelium growth in pathogenic fungi that is similar to *B. cinerea* at high concentrations (range of 0.5–20 mM). They also tested different iron salts and found that those with bromide, chloride and sulfate anions showed the best antifungal activity. In particular, the addition of an ammonium counterion to the sulfate moiety contributed to the inhibition of mycelium growth in the pathogenic fungus *Eutypa lata,* an ascomycete like *B. cinerea*.

Therefore, taking these results from previous works into account^27,28,30,53^, we decided to test TPPS, a molecule that presents four external sulfonate groups linked by a tetrapyrrole ring. Indeed, TPPS with an ammonium counterion could be an excellent antifungal candidate. Moreover, it was demonstrated that TPPS remains negatively charged in a large array of chemical environments, even under acidic pH and does not aggregate in media, allowing it to diffuse through cell walls and membranes more easily^29,30^. Therefore, this PS was thought to be an excellent candidate for APDT.

Under white light, TPPS at a very low concentration (MFC = 1.5 µM) induced a severe inhibition of *B. cinerea* mycelium growth which led to death. We did not succeed to rescue the mycelium after this treatment. To our knowledge, there is little to no information available on the fungistatic or fungicidal effect of light-activated photosensitizers on *B. cinerea*^54^. The first step was to investigate whether the anionic porphyrin was able to induce any changes in the mycelium structure. In the previous study, it was shown that the structure of the *B. cinerea* hyphae changed after treatment with antibiotics, eugenol, FeSO_4_ and tea tree oil^50,53,55,56^. Moreover, it has been shown that stressed mycelium often produces spores and could show altered cell elongation^57^. According to our data, TPPS also induced a structural change on the fungus. More specifically, the structure of the PS-treated fungus was thinner than the structure of the control. The treated mycelium produced spores implying that the fungus was under stress. Therefore, it was of interest to localize TPPS inside the cells. In tobacco plant cells, TPPS was also tested at 3.5 µM and it was the most effective porphyrin PS to induce cell death under a short light period of 5 h and it was proven to mainly localize in the cell wall^30,58^. Thus, a similar localization of TPPS was expected in the fungal cells. The multi-layer fungal cell wall is enriched in neutral sugars and proteins and poor in chitin and uronic acids, suggesting a neutral global charge of the cell wall^59^. Nevertheless, the *B. cinerea* cell wall composition and it’s global charge remain controversial. It was hypothesized that this neutrality could allow TPPS to cross the fungal cell wall which is completely different from the tobacco cell wall^30,59,60^. Further analyses, such as the H_2_O_2_ content, MDA, MTT, total thiols and microscopy analysis, confirmed fungus stress to the point of dying. Our findings proved that TPPS, due to its characteristics, could be a valid alternative to classic fungicides^29^.

From our previous studies, 50 µM TPPS was our reference for plantlets grown in vitro from seeds: Arabidopsis and tomato^27,28^. In this new study, we changed our plant model: grapevines that are obtained from clones, to establish a pathosystem with *B. cinerea*. Comparing with the other plant species, the grapevine varieties did not grow optimally at root apparatus level in the presence of 50 µM TPPS. Thus, the concentration was reduced to 12.5 µM TPPS for all the chosen varieties: Chardonnay, Merlot and Sauvignon. The choice of these three varieties was due to their different susceptibility to *B. cinerea*^42,43^. Furthermore, 12.5 µM TPPS did not induce any phenotypical nor biochemical modification of the three grapevine plantlets. In addition, this TPPS concentration was approximately ten times higher than the minimal concentration inhibiting the mycelium growth (1.5 µM); thus, the strategy presented in this article could work against pathogens without altering plant growth and development.

The final aim was to demonstrate TPPS efficiency against *B. cinerea* in 2-month-old infected grapevine leaves to validate the hypothesis of mimicking a pathosystem. The in vitro cultures and artificial system demonstrated the potential of our strategy. ESEM images from infected leaves, of the three grapevine varieties, after 72 h treatment confirmed that these leaves infected with *B. cinerea* pre-treated with 50 µM TPPS were similar to that of the control. For the Sauvignon variety, the presence of spores on infected leaves confirmed that it is more sensitive to the fungus infection than the other two varieties^42,43^. This is also confirmed by the measure of the total thiol content, considered as a primary and strong defense to infection^61^.

In conclusion, TPPS was able to kill the pathogen *B. cinerea* without harming the grapevine leaves in vitro. Moreover, this molecule does not produce any biochemical nor phenotypical changes on the plantlets grown in vitro*.* These preliminary experiments carried out are indeed promising and, in the future, experiments could be done in a greenhouse and in fields to determine the real potential and efficacy of TPPS against plant pathogens. The results and findings presented herein are also very encouraging because the photodynamic treatment has been developed using a low concentration of PS. Therefore, we show that APDT can be used for the struggle against phytopathogens in the agronomic practices as the PS is effective against plant pathogens and exhibits non-toxic side effects toward plants.

## Material and methods

### Photosensitizer

5,10,15,20-(tetra-4-sulfonatophenyl) porphyrin tetra-ammonium (TPPS) was purchased from PorphyChem (Dijon, France). The stock solution (1 mM) was prepared in distilled water and kept in the dark at room temperature for 2 weeks.

### *Botrytis cinerea* culture

The *B. cinerea* strain (UBOCC-A-117017) used in this study was isolated from infected tomatoes and provided by Dr Weill (Université de Bretagne Occidentale, Brest, France). The culture was maintained on potato dextrose agar (PDA). The growth curve of *B. cinerea* was performed as follows: a plug (0.6 cm diameter) of 2-week old *B. cinerea* mycelium was placed in the middle of plates containing PDA medium supplemented with or without TPPS in the concentration range 0.5–3.5 µM. TPPS was added to the PDA medium just after autoclaving (120 °C, 20 min). Plates were incubated at 22 °C, either in the dark or subjected to a photoperiod of 16 h (Osram large spectrum white lamp: photon flux density of 120 μmol m^−2^ s^−1^). Daily measurements of the diameter of the fungus were performed and reported to draw the growth curves.

### Grapevine clone culture

Chardonnay (clone 7535) was provided by Pr. Clément (Université de Reims, Champagne-Ardenne, France). Sauvignon (clone 379) and Merlot (clone 373) were provided by the Institut Français de la Vigne et du Vin (Bordeaux, France). Intermodal explants of grapevine were dissected and placed in glass tubes or jars containing half Chée and Pool medium, and 2% (w/v) sucrose-solidified medium (pH 5.9) for 2 months. TPPS was added to the medium after autoclaving. Chée and Pool medium was purchased from Duchefa Biochemistry (Haarlem, Holland). The cultures were then exposed to white light (Osram large spectrum white lamp: photon flux density of 120 μmol m^−2^ s^−1^) for 16 h and the temperature was maintained at 24 °C.

### Infection of young leaves with *B. cinerea*

Two-month-old grapevine leaves and four-day-old mycelium plug (0.6 cm diameter) were used to perform the experiment. Mycelium discs were firstly incubated in 12.5 or 50 µM TPPS in the dark and gently stirred for 8 h at 22 °C. Fungus discs were then placed on the upper leaf epidermis of the grapevine (Chardonnay, Merlot and Sauvignon) and were left for at least 72 h under white light (Osram large spectrum white lamp: photon flux density of 120 μmol m^−2^ s^−1^). The co-cultures were monitored daily and photographed using a Leica stereomicroscope.

### MTT assay

A 3-[4,5-dimethylthiazol-2-yl]-2,5-diphenyltetrazolium bromide (MTT) assay was carried out on the fungus mycelium, that was treated with TPPS or not, and had grown for 4 days under photoperiod. The MTT assay was performed to quantify the mitochondrial activity of mycelial cells. The fungal samples were frozen in liquid nitrogen and were grounded to a powder. 1 mL of 0.1% (m/v) MTT solution was added to 150 mg of powder. Samples were left in the dark and stirred for 3 h at room temperature. The suspension was centrifuged at 4000×*g* for 10 min. Supernatant was discarded and 1 mL of isopropanol was added to the pellet. Samples were vortexed and centrifuged at 4000×*g* for 10 min. Absorbance was read at 590 nm.

### Determination of malondialdehyde content

Approximately 150 mg of fresh or frozen fungal material was grounded in liquid nitrogen. 1.5 mL of 20% (w/v) TCA was added into the powder. The mixture was centrifuged at 13,000×*g* at 4 °C for 20 min. The supernatants were analyzed for their malondialdehyde (MDA) content as described by Issawi et al.^28^.

### Hydrogen peroxide quantification

The measurement of Hydrogen peroxide (H_2_O_2_) was performed according to Guillaumot et al. 2016^27^. 1 mL of extraction buffer (50 mM, pH 7.8) was added to fresh samples such as mycelium, plantlets or in vitro detached leaves. The composition of the extraction buffer added to the mycelium samples was 1 mM EDTA, 1% (w/v) PVP, 10% (v/v) glycerol and 1 mM DTT. For the plantlet and the leaf samples, the extraction buffer was almost identical, however PVPP was used in the buffer, instead of PVP. All samples had been frozen in liquid nitrogen, prior to being grounded into a powder. Homogenates were centrifuged at 13,000×*g* at 4 °C for 20 min. 335 μL of 0.1% titanium III sulfate (v/v) was dissolved in a solution of 20% (w/v) H_2_SO_4_ and this solution was added to supernatants. Absorbances were read at 415 nm and H_2_O_2_ levels were expressed as nM g^−1^ protein. Protein concentration was determined by Bradford assay using BSA as standard (Bradford, 1976)^62^.

### Total thiol assay

Approximately 100 mg of fine powder has been obtained from the samples (fungus, plantlets or in vitro detached leaves). After grinding in liquid nitrogen, 1 mL of 0.2 N HCl was added to the powder. A centrifugation at 13,000×*g* for 20 min was performed. Afterward, 500 μL of the supernatant was neutralized with 400 μL NaOH (0.2 M) and 50 μL NaH_2_PO_4_ (0.2 M). 700 μL of 0.12 M NaH_2_PO_4_, 6 mM EDTA and 0.1 mL of 6 mM dithiobis-2-nitro-benzoic acid (DTNB) was added to 200 μL of extract. A standard calibration curve was prepared by replacing the extract with 0, 5, 10, 25 and 50 μg/mL glutathione solutions (total volume 1 mL). Absorbance at 412 nm was read 5 min after the addition of glutathione or extract.

### Environmental scanning electronic microscopy

Environmental Scanning Electronic Microscopy (ESEM) was performed on *B. cinerea* mycelium and in vitro detached leaves after fungus infection. Mycelium grew on plates supplemented or not with TPPS for 4 days under 16 h photoperiods and was examined under Environmental Scanning Electronic Microscope (ESEM Quanta 450, Felmi-ZFE, Graz, Austria). Sizes of hyphae or branching filaments that constitute the mycelium of the fungus, were measured from ESEM pictures.

Two-month-old healthy grapevine leaves and leaves infected with *B. cinerea* pre-treated or not with TPPS were examined under ESEM.

### Confocal microscopy analysis

Mycelium was cultivated for 4 days on media containing 3.5 μM TPPS in the dark. Data acquisition with a LSM510META Zeiss confocal microscope (Carl Zeiss France, Marly-le-Roi, France) was performed under the spectral acquisition mode for TPPS localization inside the mycelium (excitation at 405 nm, emission detected at 640 nm) and under the channel mode for examination of the mycelium.

### Statistical analysis

All biological experiments were performed at least three times independently. Results were expressed as a mean ± SD (Standard Deviation). The data were analyzed by one-way ANOVA using the PAST free software.
